# Health-Related Quality of Life, Family Conflicts and Fear of Injecting: Perception Differences between Preadolescents and Adolescents with Type 1 Diabetes and Their Mothers

**DOI:** 10.3390/bs11070098

**Published:** 2021-07-06

**Authors:** Marta Tremolada, Maria Cusinato, Sabrina Bonichini, Arianna Fabris, Claudia Gabrielli, Carlo Moretti

**Affiliations:** 1 Department of Development and Social Psychology, University of Padua, 35131 Padova, Italy; s.bonichini@unipd.it; 2 Pediatric Diabetes Unit, Department of Women’s and Children’s Health, Azienda Ospedale Università di Padova, 35127 Padova, Italy; maria.cusinato@aopd.veneto.it (M.C.); ariannafabris88@gmail.com (A.F.); claudia.gabrielli@aopd.veneto.it (C.G.); carlo.moretti@aopd.veneto.it (C.M.)

**Keywords:** adolescents, diabetes type I, quality of life, family conflicts, fear of injecting

## Abstract

Good management of diabetes requires at the same time self-regulation behaviour and a balanced involvement of family components. This cross-sectional study’s aims were: understanding fear of injections and perceptions of family conflicts in preadolescents and adolescents with type 1 diabetes mellitus and their mothers, comparing their perceptions, and identifying the risk factors impacting patients’ quality of life. Eligibility criteria were: treatment for diabetes mellitus type I, currently aged 10–18 years, attending the hospital for annual hospital follow-ups. Exclusion criteria were: intellectual disabilities, inability to complete questionnaires alone and neuropsychiatric illness with active pharmacotherapy. The study design was cross-sectional. Participants were one hundred and two patients (Mean age = 14.6, SD = 2.4; age range = 10–19 years; Females = 52 and Males = 50) and their mothers (Mean age = 46.9, SD = 6.2, age range = 27–63 years), who filled in self and proxy-report questionnaires (N total= 204). The results showed that 20% of patients and 14.7% of their mothers reported clinical scores for fear of self-injection and blood testing. The mothers reported lower fear of injecting and higher family conflicts compared with the patients. Age, fear of injecting and family conflicts were significantly associated with patients’ quality of life perceptions. Clinical considerations and recommendations are given based on the empirical results.

## 1. Introduction

Type 1 diabetes (T1D) is one of the most common chronic diseases among the pediatric population. The worldwide annual incidence is 98,200 (128,900) new cases in childhood and adolescents [1]. Type 1 diabetes requires a demanding and time-consuming treatment regimen that includes blood glucose monitoring, multiple insulin doses, carbohydrate counting and physical activity [2]. Good management of diabetes requires at the same time self-regulation behavior [3] and a balanced involvement of family components [4,5,6]. Specifically, a sharing of diabetes-related tasks and responsibilities between parents and pediatric patients is associated with better diabetes outcomes [7]. A collaborative partnership with open communication and emotional support by parents positively intervenes in diabetes management [8]. Parental responsibility decreases with time, and adherence and metabolic control can deteriorate, especially during preadolescence and adolescence [9]. In this period teenagers have to balance their dependence on their parents and their desire to acquire higher autonomy in diabetes management [10]. During the transition from childhood to adolescence, patients experience difficulty in managing their behavior, and in some cases family conflicts, diabetes-related distress and poor psychological outcomes could emerge [11,12].

Indeed, children and adolescents with T1D are more likely to suffer from depression, anxiety and psychological distress with negative impacts on their quality of life perceptions [13,14]. In particular, adolescents with T1D experience more externalized problems and report more family conflicts, with an important impact on glycemic control [15]. Family conflicts can be associated with youth diabetes adjustment in a direct or indirect way [16]. The first way includes the open expression of anger concerning diabetic management. The second way includes different types of conflicts not related to diabetes adherence but associated with having T1D [17]. Existing research shows that family conflicts are associated with lower quality of life and higher glycated hemoglobin (HbA1c) [15].

Moreover, another aspect that significantly impacts on glycemic control and psychological distress in youth with type 1 diabetes is fear of self-injecting [18,19]. Research shows that fear of needles is common among patients with T1D [20,21,22]. More intense needle phobia is associated with poor management, such as higher HbA1c level, rare glucose monitoring [20] and higher risk of long term complications [23]. The parents of children with type 1 diabetes also experience fear and distress during insulin injection and glucose testing procedures. Previous research shows that 13.6% of mothers reported needle phobia and distress during procedure after the diagnosis as well [24]. To the best of our knowledge few studies analyze this aspect among parents of T1D patients [24,25].

Many factors influence the quality of life (QOL) of patients with T1D: sociodemographic, personal, diabetes-specific and family factors [16,26,27,28]. Children with T1D report higher quality of life than adolescents [29]. Moreover, some specific diabetic variables affect quality of life: good metabolic control and intensified insulin therapy are associated with better quality of life [30]. Conversely, fear of insulin and injection constitute risk factors for poor glycemic control, psychological maladjustment and poor general well-being [18]. In addition to medical and personal factors, family variables impact on quality of life, emphasizing the role of quality of family interactions, parental monitoring and family communication. In particular, intrusive parental involvement is associated with lower quality of life in adolescents with T1D and with greater family conflicts [15,31]. Adolescents, in a qualitative study, reported that parental intrusiveness, blame and lack of understanding are connected with family conflict [32].

The purpose of the current study is to explore health-related quality of life in preadolescents and adolescents with T1D and their mothers, taking into account sociodemographic variables, such as age and gender; diabetes-specific variables, such as fear of self-injection and self-testing; and family factors, such as family conflict. The present study has three goals. The first goal is to assess fear of injections and the level of perceived family conflicts in a group of T1D pediatric patients and their mothers. The second goal is to investigate the possible differences between the patients and their mothers regarding fear of injection and perception of family conflicts. The third purpose is to test possible differences between preadolescents and adolescent patients in quality of life, perception of family conflicts and fear of injections. The last goal is to identify patients’ (age, gender, HbA1c, fear of injecting, family conflict) and mothers’ characteristics (age, years of schooling/level of education, family conflict, fear of injecting) that may impact on patients’ quality of life.

## 2. Materials and Methods

### 2.1. Study Design

The design of the study is cross-sectional. It is a type of observational study that analyzes data from a population, or a representative subset, at a specific point in time—that is, cross-sectional data. In this case the preadolescents and adolescents with Type I diabetes were deeply studied. 

### 2.2. Setting and Procedure

The sample enrolment and questionnaire administration were conducted during scheduled diabetic visits by a clinical psychologist. The research project was presented to preadolescent and adolescent patients and their parents, explaining the principal aims of the project and the self-reported questionnaires. Parents signed the informed consent at the hospital. The recruitment period lasted 2 months (during winter) according to the hospital ward and Ethical Committee. All subjects were informed of the confidentiality of data and that they could withdraw from the study at any moment. The study followed the Declaration of Helsinki (Italian law 196/2003, UE GDPR 679/2016) and it was approved by the hospital’s institutional board of review (IRB) of the University of Padua. The questionnaires were given to patients and parents during their diabetic visit in a quiet room of the Clinic and were completed on this occasion, with the clinical psychologist providing assistance. Prior to the present study, a pilot study was conducted in a South Tirolean group of pediatric diabetic patients, with the adopted questionnaires showing good psychometric properties [26].

### 2.3. Participants and Study Size

Participants of our study were enrolled at the Department of Woman and Child’s Health of University Hospital of Padova. One hundred and two patients and their mothers (N tot = 204) were recruited during periodic follow-up visits at the Diabetes Unit. Eligibility criteria were: treatment for T1D, patient’s age between 10 and 19 years and a T1D diagnosis at least 6 months prior to data collection. Exclusion criteria were: intellectual disabilities, inability to complete questionnaires alone and neuropsychiatric illness with active pharmacotherapy. We obtained this sample size with all contacted patients throughout two months that accepted to participate to the study. It seemed representative of pre-adolescent and adolescent diabetic patients in the one-center Clinic of Padua.

### 2.4. Variables

The outcomes were the following: health-related quality of life in patients, family conflict and fear of injecting both in patients and their mothers. Exposures were: time from diagnosis, all patients affected of diabetes mellitus type I. Predictors were: current age, glycemic control. Potential confounders or effect modifiers were: parental relationship, economic condition and type of education. The quantitative variables were means of the scoring of the several indexes, Additionally, the groupings chosen were two age ranges in pediatric patients representative, respectively, of preadolescents and adolescents following the developmental stages. Another grouping was composed of mothers and their children to compare family conflicts and fear of injecting.

### 2.5. Measures

#### 2.5.1. Diabetes Quality of Life for Youth-Short Form (DQOL-SF)

The DQOL-SF [30,31] consists of 18 items scored using a five-point Likert scale (from 0 = “never” to 4 = “all the time”), administrable from 10 years of age. Higher scores indicate a poorer quality of life. In this study it showed a good internal consistency, both for the global scale (α = 0.7; N item = 18) and for the two subscales: impact of diabetes on daily life (α = 0.6; N item = 11) and worries about diabetes (α= 0.9; N item=7). Examples of items are as follows: *“You feel you have limited social relationship”* (Item 4), *“How often do you worry whether you will have complications”* (Item 18), respectively, belonging to two subscales. The Italian validation was performed on a sample of children aged 8–18 years, showing good psychometric properties [26].

#### 2.5.2. Revised Diabetes Family Conflict Scale (DFCS-R)

The DFCS-R was completed by pediatric patients and their parents [17,33]. It includes 19 diabetes management tasks to assess the degree of family conflict. The self-report score is on a three-point Likert scale (from 1 = never argue to 3 = always argue). Higher scores indicate higher levels of conflicts. In this study internal consistency was good in both versions: α = 0.96 for child/adolescent version and α = 0.98 for parent version. An example of items included is: *“During the past month, I have argued with my parent(s) about Remembering to check blood sugars”* (Item 3) for children/adolescents’ version.

#### 2.5.3. Diabetes Fear of Injecting Questionnaire (D-FISQ)

Children/adolescents (self-report) and parents (proxy-report) completed D-FISQ [22] to assess fear of self-injection and blood glucose testing. This questionnaire is composed of 30 items, divided into two subscales: fear of self-injecting, fear of blood glucose testing. The score is attributed on a four-point Likert scale (from 0 = never to 3 = always). A score ≥ 6 indicates a clinical fear of needles. In our study internal consistency was: fear of self-injection, α = 0.8 for the children/adolescents version, α = 0.88 for the parent; fear of blood glucose testing, α = 0.9 for the children/adolescents version, α = 0.88 for the parent version. Examples of items for patient version are: “*When I have to inject myself I become restless*” (Item 1); “*When my mom/dad injects me she/he feels afraid*” (Item 20), concerning self-injection subscale. Examples of items for parents’ version are: “*When my child injects himself He/she feels tense*” (Item 2), concerning injection subscale; “*When I have to test my child’s blood glucose I become restless*” (Item 26), concerning blood glucose testing subscale.

#### 2.5.4. Diabetes Information

The diabetes-related information was derived from the clinical sheets. Several pieces of information were collected such as the time length form the diagnosis, participants’ age at type 1 diabetes onset, insulin regimen and glycemic control (measured as the most recent HbA1c value).

### 2.6. Statistical Methods and Bias

Descriptive measures of central tendency and variability were computed for all the dependent variables. The distribution of the all the variables was not normal (*p* < 0.01 in Shapiro tests), so non-parametric analyses were run. In order to identify possible differences between patients with T1D and their mothers regarding fear of injections (assessed by *D-FISQ* self-report and proxy-report) and perception of family conflicts (assessed by *DFCS-R* with children and parent version), two *Z* Wilcoxon test for dependent samples were performed. To test possible differences between preadolescent and adolescent patients in all variables (quality of life, perception of family conflicts and fear of injections), we performed two different analyses. At first, we divided our sample into two groups: preadolescents (10–14 years) and adolescents (15–19 years). Second, we performed a *U* Mann–Whitney test for independent samples to compare preadolescents’ and adolescents’ scores. Spearman’s correlations were run to identify the possible associations between the variables.

Statistical analyses were carried out using IBM Corp SPSS Statistics 22.0 (Armonk, NY, USA). A *p*-value < 0.05 was considered statistically significant. We adjusted the p value because of multiple testing and to avoid a type 2 error dividing the p-value with the number of comparisons. In this sense we accepted only results with a *p* ≤ 0.01.

## 3. Results

### 3.1. Descriptive Data of Participants

All the patients recruited and their mothers agreed to participate to the study, obtaining a total of 204 participants. Patients’ mean age was 14.63 (SD = 2.43); 52 were female and 50 were male. Mothers’ mean age was 46.94 (SD = 6.2 range 27–63). Of the included mothers 1% had only graduated primary school, 39.2% had a middle school diploma, 42.2% had a high school diploma, 13.7% had a university degree and 3.9% had a postgraduate degree. Table 1 and Table 2 show the socio-demographic information of patients and their mothers.

### 3.2. Quality of Life and Family Conflicts of Patients and Their Mothers Compared with Norms

Descriptive analysis of fear of injecting and of blood control, family conflicts and quality of life are reported in Table 3. Assuming a score ≥ 6 indicated a clinical level for fear of needles, as indicated from D-FISQ authors, we found that 20% of patients (*N* = 21) and 14.7% of their mothers (*N* = 15) reported clinical levels of fear according to D-FISQ global scores. Regarding perceived family conflicts, patients reported a mean score of 17 (SD = 10.6), while mothers reported a mean of 20.97 (SD = 11.6). The conflict ratings attested to a low level both for pediatric patients and for their mothers, considering that the scale ranged from 19 (=no conflict) to 57 (=high level of conflict). Health-related quality of life for our patients showed a mean score of 16.3 (SD = 7.2). Higher scores indicate a more negative impact of diabetes and poorer QoL, and lower scores indicate better QoL. These pediatric patients reported lower quality of life perceptions.

### 3.3. Comparison between Patients’ and Their Mothers’ Reports on Patient’s Fear of Injecting and Family Conflicts

**Figure 1:**
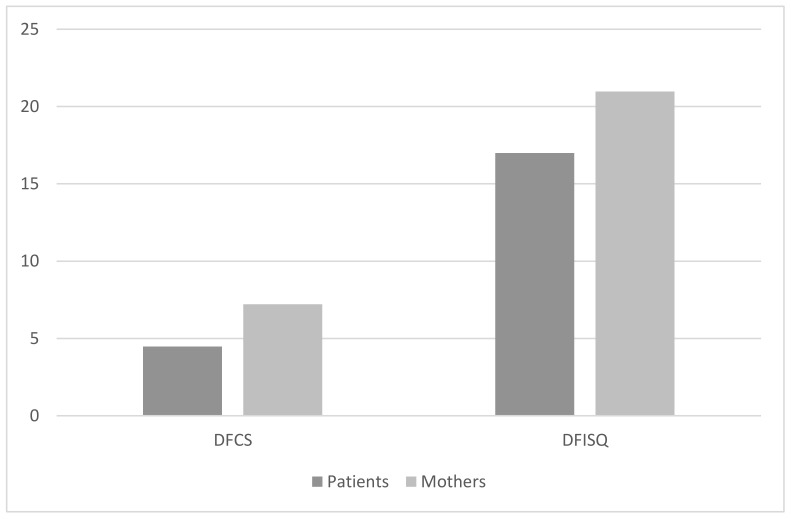
Differences between patients and their mothers regarding fear of self-injecting and family conflicts. Legend: DFISQ: diabetes fear of self-injecting questionnaire; DFCS = diabetes family conflict scale.

A Z Wilcoxon test for dependent samples was run to identify possible differences between patients’ self-reports and mothers’ proxy reports on patients’ means of fear of injections. A significative difference between means of fear of self-injections were found in 99 out of 102 patient–mother couples (Z = −2.6, *p* = 0.008), with patients reporting a lower mean of fear (Mean = 4.5, SD = 6.7) compared with that reported by their mothers about their sons/daughters (Mean = 7.2; SD = 9.6). No difference was shown for the fear of self-testing (*p* > 0.05). Another Z Wilcoxon test for dependent samples was run to identify possible differences between patients’ and mothers’ family conflicts in 101 of 102 patient-mother couples. The results showed a significative difference between mothers and their daughters/sons (Z = −3.6; *p* = 0.0001), with patients declaring a lower score in their family conflicts (Mean = 17; SD = 10.6) compared with that reported by their mothers (Mean = 21; SD = 11.6) (Figure 1).

### 3.4. Factors Associated with Patient’s Quality of Life

We ran Spearman’s correlations to identify the possible significative associations between patients’ and mothers’ socio-demographic (age, gender) and diabetic factors (HbA1c, time in years from the diagnosis) and patients’ quality of life. Only patients’ age was identified as a factor associated, respectively, with patients’ quality of life (rho = 0.2, *p* = 0.013), especially the subscale of Worries (rho = 0.3, *p* = 0.005) and with mothers’ family conflict (rho = 0.3, *p* = 0.001). Mothers’ age was significantly and negatively associated with her perceived family conflict score (rho = −0.3, *p* = 0.004). Length of diagnosis was significantly associated with patients’ worries about their quality of life (rho = 0.2, *p* = 0.03), while glycemic control did not show any significant association (rho = 0.05; *p* = 0.6), and likewise with gender (rho = 0.02, *p* = 0.8). We adjusted the p value because of multiple testing and to avoid a type 2 error dividing the p-value with the number of comparisons. In this sense we accepted only results with a *p* ≤ 0.01 (Table 4).

We performed a *U* Mann–Whitney test for independent samples to compare preadolescents’ and adolescents’ scores along their quality of life perception. We obtained a significant difference in quality of life perception (U = 911.5; *p* = 0.010), with preadolescents showing lower scores (a best quality of life) (Mean ranks = 43.5) than adolescents (Mean ranks = 58.6).

### 3.5. Factors Associated with Mother’s Family Conflict Score

Regarding the correlations between the fear of self-injecting/self-testing, family conflict score and quality of life, the findings showed that patients’ global quality of life was significantly associated with their own perceived fear in self-testing and self-injecting (rho = 0.3; p = 0.0001) and with their reported family conflict score (r = 0.30 *p* = 0.002). Mothers’ fear of injecting/testing was significantly associated with the family conflict score reported by them (r = 0.5; *p* = 0.0001) or by their children (r = 0.3; *p* = 0.005). We adjusted the p value because of multiple testing and to avoid a type 2 error dividing the p-value with the number of comparisons. In this sense we accepted only results with a *p* ≤ 0.01.

## 4. Discussion

This pilot observational and cross sectional study examined health-related quality of life, family conflicts and fear of injections in preadolescents and adolescents with Type 1 Diabetes (T1D) and their mothers in an Italian district cohort. The main results showed that 20% of patients and 14% of their mothers reported clinical scores of fear of injection and fear of self-testing of blood glucose. This result is consistent with the literature, which underlines the same percentages of fear of needles in patients with T1D [22]. Even if this fear seemed to be not so common in pediatric patients, and only a few studies analyzed this aspect in pediatric patients with T1D [19,20,21,22,23], an Italian study [26] showed that parental fear about their children’s self-injection of insulin was identified as a key element impacting on externalizing/internalizing symptoms and on worries about the illness.

Compared to other studies on this topic [20], our results did not show an association between HbA1c and needle phobia. Other studies in fact highlighted the association between intense needle phobia and less frequent glucose monitoring and insulin corrections that expose patients to higher HbA1c and risk of long term complications [20]. The absence of association between higher needle phobia and higher HbA1c in our cohort could be due to high mean levels of HbA1c recorded among the studied participants and a quite similar distribution of this parameter. This is a unique single center study and the results are preliminary, so other data are necessary to generalize this result.

It might be useful in future research to compare scores of needle phobia with the novel glycemic metrics derived from the use of Continuous or Fast Glucose Monitoring (CGM of FGM) systems [34,35]: TIR (“time in range”: the time the patient spends in the optimal glycemic interval of 70-180 mg/dL), TAR (“time above range” of 180 mg/dL) and TBR (“time below range” of 70 mg/dL). These core metrics can express better than HbA1C glycemic variability, acute excursion of glucose change and severity of hypo and hyperglycemia, all clinical situations that could require a more intensive self-monitoring of blood glycemia or insulin self-administration, which might instead be voluntarily omitted due to the level of needle phobia.

Another key finding in our study showed that the patients with more years from the diagnosis reported more worries related to their health-related quality of life, stressing how the chronic condition of facing and managing this illness daily may worsen their emotive difficulties.

As observed in previous research, risk factors associated with a lower quality of life in T1D pediatric patients could be the following: adolescent age, which is associated with lower quality of life perceptions also in not-clinical population [28]; the higher presence of needle fear very common in patients with T1D [18]; and family conflicts, with the important role of family interactions and communication [30].

Assessing mother–patient agreement on their questionnaires scoring, mothers and patients highlighted significant differences in fear of injection and family conflict scores: patients reported significantly lower levels regarding these two dimensions compared with their mothers. Previous research reported higher level of family conflict in pediatric patients than in parents [36,37]. Only one study reported not significant discrepancies in this dimension between young adults and their parents [38]. This result showed differences in perceptions. Probably mothers seemed to perceive lower ability in their sons/daughters to self-inject and self-test, and the strength needed to maintain the daily balance was so great that they perceived more conflict relationship with their sons/daughters.

Finally, mothers’ fear of injecting was associated with their own and their son’s family conflict scoring, showing how these two constructs should be taken into consideration in the future studies with a larger cohorts.

## 5. Limitations, Future Implications and Clinical Suggestions

The current study provides useful evidence in the research and clinical practice concerning fear of injections, family conflicts and quality of life in adolescents and preadolescents with type 1 diabetes. However, there are some limitations that can be addressed in future research. First, the present study employed a cross-sectional design, so future longitudinal studies should be added to observe variation of fear of injection and family conflict during chronic management of diabetes. Second, our findings are based on small sample size; for this reason, results cannot be generalized. Our data are not distributed normally, so analyses are limited. Future research could involve different centers to obtain major data. Moreover, the present study is based on mothers and youth assessments with the exclusion of fathers, a fact that constitutes the major limitation of the present study. According to an ecological framework, future research should take into account fathers’ perceptions of diabetes, as well as the perceptions of siblings of patients with T1D in order to better investigate family functioning and concerning variables, such as quality of family dynamics. Other qualitative data would be useful to better understand family and psychological variables. This study includes only self-report measures that expose results to limitation due to social desirability. Finally, the present study takes into account only HbA1c as a medical index; future research should also include TIR (Time in Range), and Time in hypoglycemia and hyperglycemia as variables of acute and chronic complications.

Despite these limitations, a number of clinical implications from this study that can improve clinical practice and guide future research could be suggested.

Fear of injection both in patients and in mothers confirms the close association between this psychological/behavioral aspect and the nature of Type 1 Diabetes treatment [39]. Therefore, preventive care and tailored psychological interventions are required to improve mental health and prevent long term complications of T1D both in young patients and in their mothers [20]. Research shows evidence to support the efficacy of hypnosis and distraction in reducing needle fear [40]. Moreover, the literature shows that relaxation techniques, such as muscular relaxation, guided imagery and deep breathing could be useful in mild needle fear [41]. Exposure-based therapy, both in vivo and non-in vivo, is recommended for individuals with high levels of needle fear, if older than 7 years of age [42]. In order to plan a needle phobia intervention it is important to take into account relevant family factors and possible past traumatic events [43].

Additional research is needed to identify effective psychological interventions regarding needle phobia and fear of injection in the pediatric population with Type 1 Diabetes.

Our findings highlight discrepancies in the perceptions of family conflicts and of quality of life between patients and their mothers and, in particular, between preadolescents and adolescents, suggesting that it could be useful to adopt screening tools to investigate family functioning, poor glycemic outcome and psychological disease during risk periods such as adolescence. Motivational interviews could be useful when facing high levels of conflict and low glycemic control [43]. Psychological intervention focused on family communication might be beneficial to motivate parents to communicate in a non-judgmental way with their children [44] and to promote family cohesion and preserve youths’ overall quality of life [15]. Moreover, it is important to take into account the parental distress of mothers facing diabetes management during the transitional period of adolescence. In fact, adolescent mental health seems to be connected with family climate across time [45].

## 6. Conclusions

In conclusion, the promotion of the psychological well-being of pediatric patients with type 1 diabetes requires close cooperation between different sources of care, such as diabetologists, dieticians, psychologists and the family context [12]. This study underlines the need to better understand the psychological characteristics of patients with T1D according to the age group in order to tailor specific psychological interventions to patients and their parents.

## Figures and Tables

**Table 1:** Socio-demographic characteristics of patients.

Socio-Demographic Variable	Range/Levels	*N*	%
Gender	Male	50	49
	Female	52	51
Age	Preadolescents (range: 10–14 years)	48	47.1
	Adolescents (15–19 years)	54	52.9
		Mean	SD
HbA1c	Range: 5.5–11.1	7.6	1
Time from diagnosis (years)	Range: 0–15	7.9	4

**Table 2:** Socio-demographic characteristics of mothers.

Socio-Demographic Variable	Range/Levels	Mean	SD
Age	Range: 27–63 years	46.9	6.2
		*N*	%
Relationship status	Married	76	74.5
	Divorced/Separated	18	17.7
	Cohabitant	6	5.9
	Single parent	1	1
	Missing	1	1
Perceived economic condition	Insufficient	8	7.8
	Sufficient	28	27.5
	Adequate	39	38.2
	Good	25	24.5
	Optimal	2	2
Level of education	Primary school	1	1
	Lower secondary school	40	39.2
	Upper secondary school	43	42.2
	University Degree	14	13.7
	Ph.D. or Master’s degree	4	3.9

**Table 3:** Mean scores of fear of injecting, family conflicts and quality of life.

Variable	Typology	Mean	SD	Minimum–Maximum
D-FISQ patients	FSI	1.3	2.3	0–12
	FST	1.6	2.6	0–13
D-FISQ patients global score	FSI + FST	2.3	4.2	0–22
D-FISQ mothers’ reports on their own experience of injecting (N = 54 for FSI and 48 for FST)	FSI	1.4	2.7	0–12
	FST	1.5	3.5	0–21
D-FISQ mothers global score	FSI + FST	3.7	5.9	0–32
DFCS patients		17	10.6	0–49
DFCS mothers		21	11.6	0–54
DQOLY		16.3	7.2	3–34

Legend: D-FISQ: Diabetes Fear of Self-Injecting Questionnaire; FSI: Fear of Self-Injecting; FST: Fear of Self Testing; DFCS = Diabetes Family Conflict Scale; DQOLY: Diabetes Quality of Life for Youth.

**Table 4:** Spearman’s correlations between socio-demographic and diabetes factors with quality of life, fear of self-injecting and family conflict in patients and their mothers.

Variable	DFISQ_Self	DFISQ_Mother	DFCS_Patient	DFCS_Mother	DQOLY	DQOLY_Impact	DQOLY_Worries
Gender	rho = 0.02 *p* = 0.8	rho = −0.01 *p* = 0.9	rho = −0.1 *p* = 0.2	rho = 0.09 *p* = 0.3	rho =0.05 *p* = 0.6	rho = −0.02 *p* = 0.8	rho = 0.1 *p* = 0.3
Patient’s Age	rho = −0.07 *p* = 0.4	rho = −0.3 ** ***p* = 0.001**	rho = −0.03 *p* = 0.8	rho = −0.09 *p* = 0.3	rho = 0.2 * ***p* = 0.013**	rho = 0.1 *p* = 0.1	rho = 0.3 * ***p* = 0.005**
HbA1c	rho = 0.009 *p* = 0.3	rho = 0.1 *p* = 0.3	rho = 0.03 *p* = 0.8	rho = 0.1 *p* = 0.2	rho = 0.05 *p* = 0.6	rho = 0.02 *p* = 0.8	rho = 0.07 *p* = 0.5
Time from diagnosis	rho = −0.06 *p* = 0.5	rho = −0.1 *p* = 0.2	rho = 0.09 *p* = 0.3	rho = 0.1 *p* = 0.1	rho = 0.1 *p* = 0.2	rho = −0.012 *p* = 0.9	rho = 0.2 * ***p* = 0.03**
Mother’s age	rho = −0.11 *p* = 0.2	rho = −0.3 ** ***p* = 0.004**	RHO = 0.1 *p* = 0.3	rho = −0.1 *p* = 0.2	rho = 0.1 *p* = 0.1	rho = 0.2 *p* = 0.07	rho = 0.06 *p* = 0.5

Legend: D-FISQ = Diabetes Fear of Self-Injecting Questionnaire; DFCS = Diabetes Family Conflict Scale; DQOLY = Diabetes Quality of Life for Youth. * = rho significant with a *p* value ≤ 0.05; ** = rho significant with a *p* value ≤ 0.01. Bold characters mean significant results.

## Data Availability

Data supporting reported results can be found requesting them to the corresponding author that archived them in a cloud file.
